# Impact of using recycled plastic particles as a partial replacement of aggregates on the mechanical performance of the eco-friendly concrete

**DOI:** 10.1038/s41598-026-56924-5

**Published:** 2026-06-16

**Authors:** Seleem S. E. Ahmad, Mohamed Khalifa Bneni, Mohammed W. Akrah, Mohammed S. Salem, Mohammed M. Beediq

**Affiliations:** 1 Faculty of Engineering, Zagazig University, Zagazig, 44519 Egypt; 2 Faculty of Engineering, University of Zawia, Zawia, 16418 Libya; 3 Institute of Science and Technology in Zawiya, Zawia, Libya

**Keywords:** Recycling of waste plastic, PE particles, Coarse and Fine aggregates, Mechanical properties, Engineering, Environmental sciences, Materials science

## Abstract

The current work contributes to the protection of natural raw resources and reduces environmental effects by recycling plastic in sustainable construction techniques. By investigating the exact transition point from a strength reducer to a functional benefit of plastic waste in the production of lightweight concrete, using coarse aggregates versus fine aggregates separately under the same curing conditions. Recycled polyethene (PE) waste, produced through thermomechanical extrusion and pelletizing, was used as a partial replacement of coarse and fine aggregates at 2.5%, 7.5% and 15% by weight. Seven concrete mixes of M25 were cast and tested for compressive strength, splitting tensile strength, and absorption rate after 7 and 28 days of curing. Findings indicated that the highest workability was achieved by replacing 15% of plastic particles with coarse aggregate (about 135 mm), while 2.5% PW had the lowest workability (about 45 mm) by replacing fine aggregate. While the highest compressive strength was obtained with a 2.5% replacement of fine aggregate, reaching about 26.22 MPa. In contrast, it decreased more severely when reaching 15% by about 21.11 MPa, compared to replacing coarse aggregates with plastic particles by about 22.44 MPa. Furthermore, substituting sand with recycled plastic particles at 2.5% replacement level resulted in the best splitting tensile strength of 1.69 MPa. It is noted that substituting coarse aggregates reduced the density more than fine aggregates, with the peak reduction rate being 3.66% for cube specimens after replacing 15% of coarse aggregates at 28 days, making it the most efficient replacement for producing lightweight concrete. All replacement levels of sand increase absorption rates by 10.4% over coarse aggregate. Therefore, these results are highly transferable to field conditions for non-structural applications such as pedestrian pathways and paving blocks, provided that workability is optimized.

## Introduction

Plastic materials are universally utilized in producing bags and bottles for our daily routines^1^. The recycling of plastic waste contributes to the production of polyethylene particles, which pose a significant environmental threat due to their resistance to biodegradation and their consumption of large areas of landfills^2^. Concurrently, the construction industry consumes immense amounts of natural resources, such as aggregate (coarse and fine aggregate), which is the primary component of concrete mass^3^. Therefore, the need has arisen for green, or eco-friendly, concrete that uses recycled materials to enhance sustainability^4^. In recent years, developments in sustainable materials have demonstrated the significant environmental benefits of recycling waste to reduce the environmental impact of industrial products in the building industry^5^. While initial attempts, such as that by Albano et al.^6^ studied the behaviour of concrete containing recycled Polyethylene Terephthalate (PET), varying the water/cement ratio to 0.50 and 0.60 and the PET content to 10% and 20%. Furthermore, the effect of PET thermal degradation in the concrete was evaluated at 200, 400, and 600 °C. Specifically, their outcomes indicated notable declines in compressive strength, splitting tensile strength, and modulus of elasticity.

Correspondingly, attempts have been made to reduce the mechanical degradation related to plastic aggregates through the addition of pozzolanic materials. For instance, Alkhraisat^7^ studied the replacement of sand by polyethene (PE) at different proportions ranging from 2.5 to 20% in mortar mixes and observed a significant decrease in compressive strength, which was later compensated for by adding silica fume. The results indicated an optimum threshold of 10% for the replacement of sand. While Tsai et al.^8^ also examined the synergy between waste PE (up to 4%) and ground-granulated blast-furnace slag (GGBFS) at different water-to-binder ratios. The results showed that the plastic content negatively affected the fresh properties, such as slump and setting time, while the long-term compressive strength was enhanced due to the secondary pozzolanic reaction of slag. Although all these studies successfully used supplementary cementitious materials to compensate for the strength loss due to plastics, they focused on mortar matrices, not concrete, leaving the distinct behaviours of coarse and fine plastic replacement in coarse aggregate techniques mostly unidentified.

In other cases, researchers have expanded the scope to study the use of bulkier waste plastics, such as Polyethylene (PE), Polyethylene Terephthalate (PET), and electronic waste (e-waste), as coarse aggregate substitutes. Akram et al.^9^ have confirmed that the use of only sliced e-plastic particles leads to a reduction in the overall strength of concrete, but after the addition of 10% fly ash, the performance was restored to the level of the reference concrete. Similarly, Ali et al.^10^ also replaced coarse aggregates with plastic aggregates up to 20% and reported improved workability but also significant reductions of 32% and 33% in compressive and tensile strengths, respectively. Adding silica fume densified the concrete matrix and decreased such losses. Nevertheless, reliance on such pozzolans leaves the actual mechanical baseline of the plastic composite undefined. This linear reduction in strength is also supported by the literature; for example, replacing coarse aggregates with PET up to 12.5% led to reductions in flexural and splitting tensile strength up to 40% and 32%^11–13^. The addition of 10%, 20%, and 30% Elec-waste coarse aggregates as partial replacements for coarse aggregates resulted in a decrease of compressive strength by about 8.97%, 27.99%, and 42.01%, respectively^14^.

Polyethylene Terephthalate (PET) has been investigated as a partial replacement of sand in concrete in many studies^15–20^. However, the literature shows very conflicting trends regarding the mechanical performance and the optimum replacement levels. Some authors have observed early decreases in compressive strength and modulus of elasticity from 10% sand replacement^21,22^, whereas others have reported mechanical improvements at lower dosages. For instance, substituting of fine aggregate with only 2% to 3% plastic waste was found to provide optimal increases in compressive strength of up to 12.55%^23,24^. On the other hand, higher replacement levels beyond 10% consistently caused severe strength degradation with reductions of up to 90.6% at a 50% replacement level^22^. The degradation is caused by a reduction in concrete density, poor interfacial bonding, incompatibility of materials, and non-uniform distribution of particles^25–27^.

## Research significance

This study aims to propose practical solutions to environmental pollution caused by plastic waste by investigating a sustainable concrete made from recycled plastic waste and by reducing the excessive use of natural resources (coarse and fine aggregates). By assessing the impact of replacing recycled plastic waste as a partial replacement (2.5%, 7.5%, and 15%) of the weight of coarse aggregates in the first group, and similar replacement ratios in the second group of sand weight. Moreover, this study addresses a gap in the existing literature by providing a direct, simultaneous comparison of coarse and fine aggregate replacement in concrete, evaluating three consistent replacement levels. It utilizes locally recycled polyethylene particles sourced from a factory in Libya, making the findings particularly relevant to the regional construction industry. Additionally, a new empirical equation for sustainable concrete incorporating recycled plastic is proposed and validated. The research quantitatively links density reduction to mechanical performance, yielding evidence-based recommendations for lightweight concrete production and distinguishing this work from prior comprehensive reviews in the field.

## Experimental program

### Materials

**Fig. 1:**
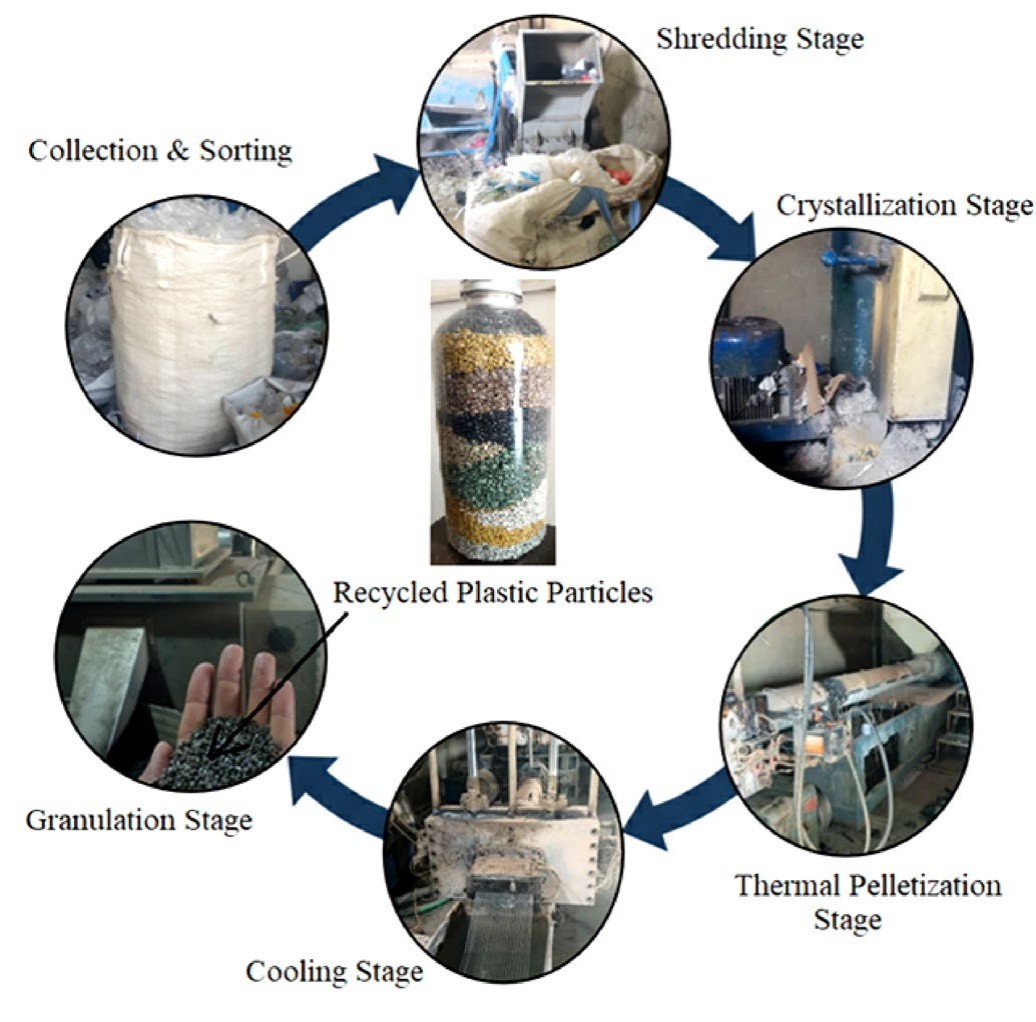
The process to transform plastic pollution into recycled plastic particles.

**Fig. 2:**
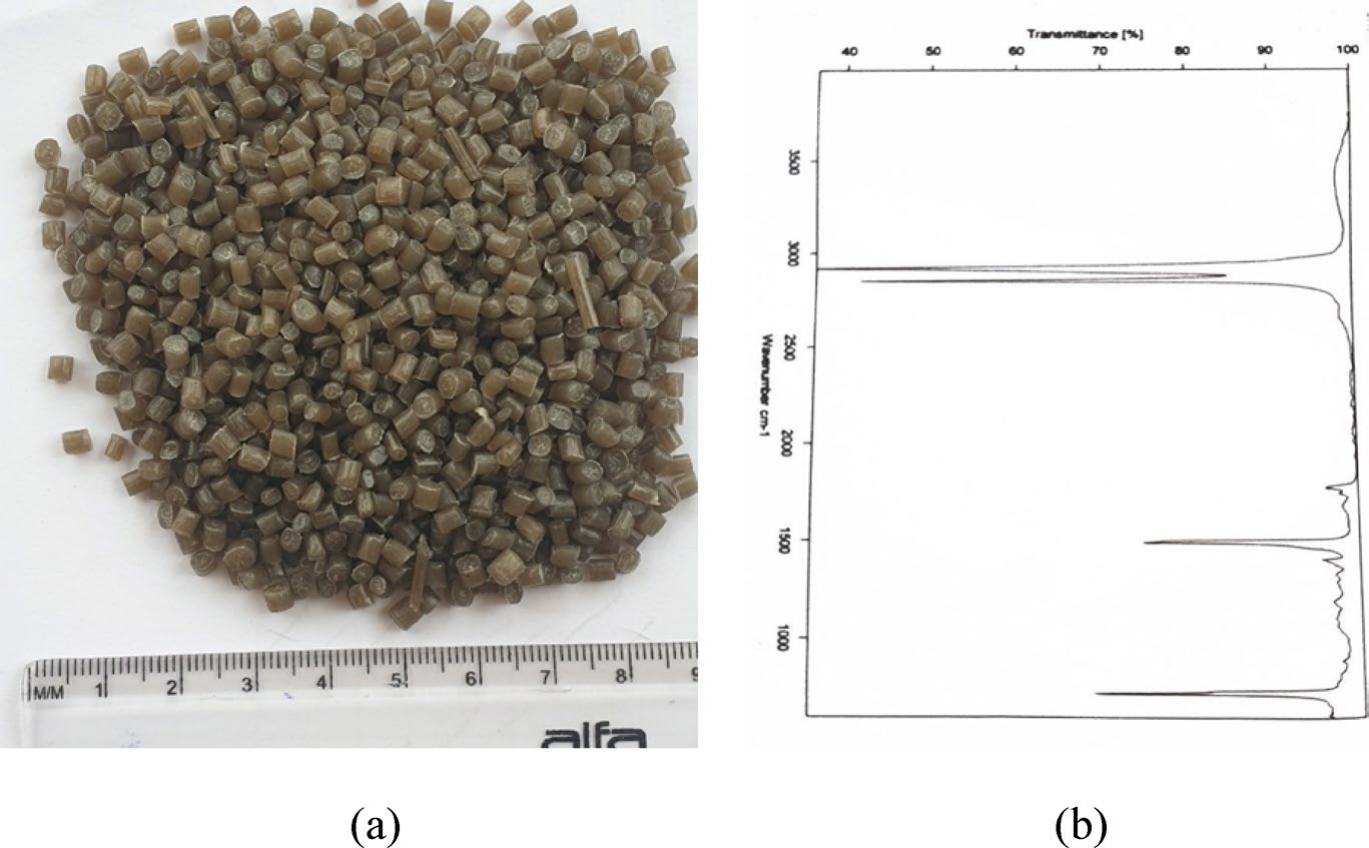
The appearance of recycled plastic waste (**a**) and the Fourier transform infrared spectroscopy (FTIR) analysis (**b**).

Recycled plastic waste was supplied from a local factory in the city of Zawiya, Libya. It collects plastic waste (bags and plastic bottles), sorting it, and then cleaning the plastic. Figure 1 depicts the process or path taken to transform waste plastics into recycled plastic granules (polyethylene) for use in the plastics industry. Then, using special machines, they are cut into small pieces or chips and heated until they melt, after which they are shaped into 2 mm-diameter wires, as seen in Fig. 2a.

**Fig. 3:**
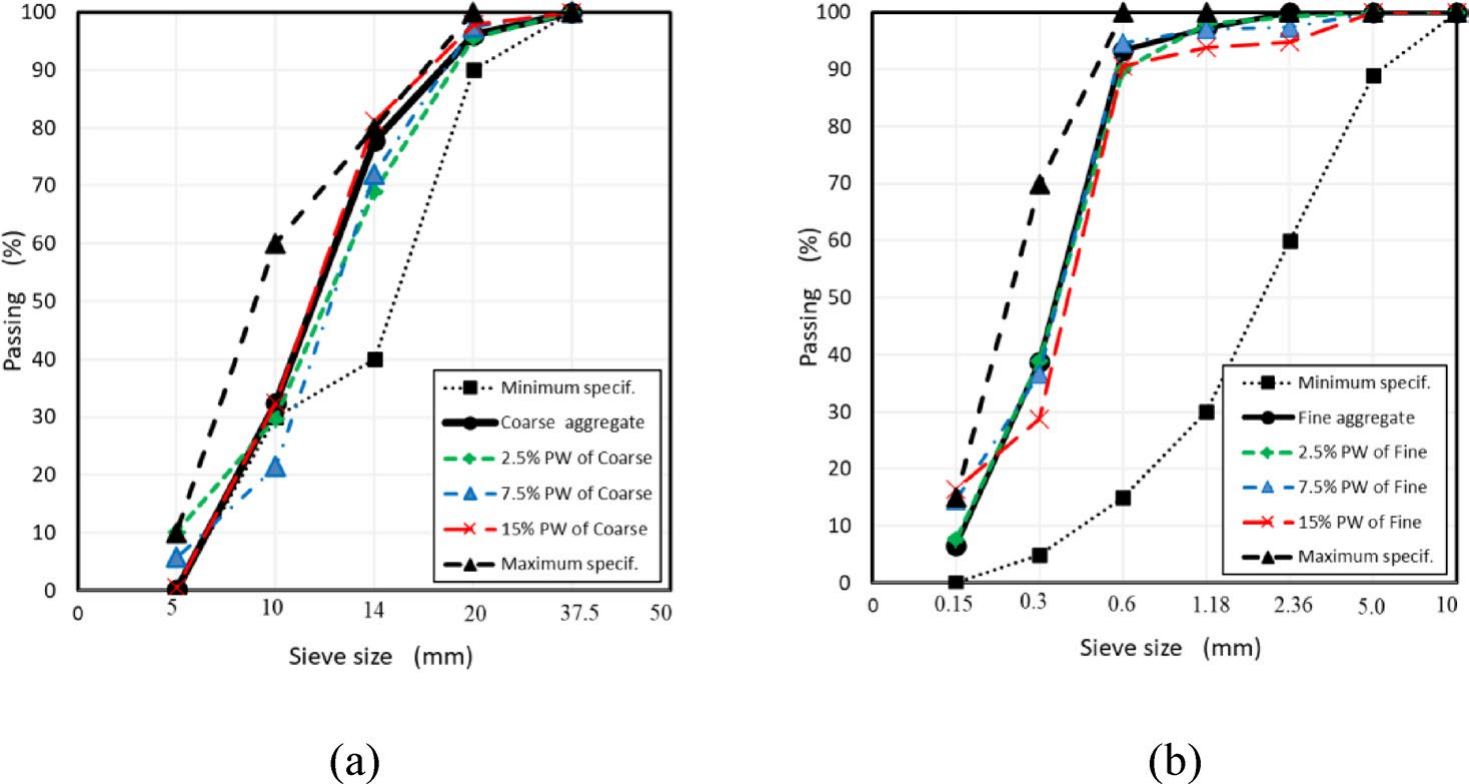
Gradation curves of (**a**) Coarse aggregate, 2.5%PW of C.A., 7.5%PW of C.A., and 15%PW of C.A., (**b**) Fine aggregates, 2.5%PW of F.A., 7.5%PW of F.A., and 15%PW of F.A.

All concrete mixes were produced of OPC (Type I-42.5 N) according to Libyan specifications, NO. 340/2009^28^. The specific gravity, initial setting time, and final setting time of the OPC were determined to be 3.14 g/cm^3^, 135 min, and 210 min, respectively. The coarse aggregate was used as angular-shaped gravel with an NMS of 20 mm. The specific gravity, bulk density, water absorption, and impact value were 2.55 g/cm^3^, 1542 kg/m^3^, 1.88%, and 21.96%, respectively, which concur with the BS 812 specifications^29^. The grading curve of coarse aggregate that complies with the specification limits of BS 882^30^ is illustrated in Fig. 3a. The fine aggregate used is sand from the city of Zliten in northern Libya, which is no larger than 5.0 mm, with a bulk density of 1609 kg/m^3^. It fulfils the standards specified by BS 812^29^, as presented in Fig. 3b.

According to laboratory tests, recycled plastic waste had a bulk density of 0.555 g/cm^3^ and a water absorption of 0.57%. The Fourier-transform infrared (FTIR) spectrum of plastic waste, which shows the highest number of polyethylene (PE) compounds, is displayed in Fig. 2b. The spectrum is characteristic of a saturated aliphatic compound with long methylene chains and agrees with the reference spectrum of polyethylene (PE). Figure 3a illustrates sieve analysis curves for mixtures that replace coarse aggregates with plastic waste at ratios of 2.5%, 7.5%, and 15% of the weight of coarse aggregates. While the plastic waste gradient was treated by replacing the sand reserved on sieve No. 50 and passing through sieve No. 30 by 2.5%, 7.5%, and 15% of the weight of sand, without affecting the granular gradient of sand, as shown in Fig. 3b.

### Mixing proportion

The current study focused on designing a mix for M25 grade concrete. For a 0% replacement level (Reference mix) and a constant water-cement (W/C) ratio of 0.57, the quantities of water-cement, fine aggregate (FA), and coarse aggregate (CA) for M25 grade concrete were determined to be 201 kg, 350 kg, 738 kg, and 1002.3 kg per cubic meter of concrete, respectively. In this research, the impacts of replacing coarse aggregate (CA) and fine aggregate (FA) with plastic waste (PW) on the concrete mixtures were evaluated in terms of workability (slump test), density, mechanical performance (compressive strength and splitting tensile strength), and water absorption capacity after 7 and 28 days of curing. Two experimental groups used recycled plastic waste to partially replace both coarse and fine aggregates. In the first group, plastic waste was used in place of coarse aggregates at weight percentages of 2.5%, 7.5%, and 15%. To maintain experimental consistency, the second group substituted the same weight percentages of plastic waste (2.5%, 7.5%, and 15%) for fine aggregates. The replacement levels of 2.5%, 7.5% and 15% were carefully selected to study the nonlinear behavior of plastic-concrete composites. The lower dose (2.5%) aims to identify the optimal filling effect, while the greater increments allow recording the transition to lightweight functional rates when replacement levels approach the structural limit. Table 1 gives details of mixtures categorized into two groups for 1.0 m^3^.

**Table 1:** Mix proportions for different trials (kg/m^3^).

Group	Name	Cement	Water	Coarse aggregate	Fine aggregate	Plastic waste
Reference	M_Control_	403.5	201	1002.3	738	0
G1	M_C2.5PW_	403.5	201	977.25	738	9.01
	M_C7.5PW_	403.5	201	927.13	738	27.05
	M_C15PW_	403.5	201	851.95	738	54.11
G2	M_F2.5PW_	403.5	201	1002.3	719.55	6.36
	M_F7.5PW_	403.5	201	1002.3	682.65	19.09
	M_F15PW_	403.5	201	1002.3	627.30	38.18

G1: Group 1, G2: Group 2, M: Mixture, C: Coarse aggregate replacement, F: Fine aggregate replacement, and PW: Plastic Waste.

### Casting, curing, and testing procedures

**Fig. 4:**
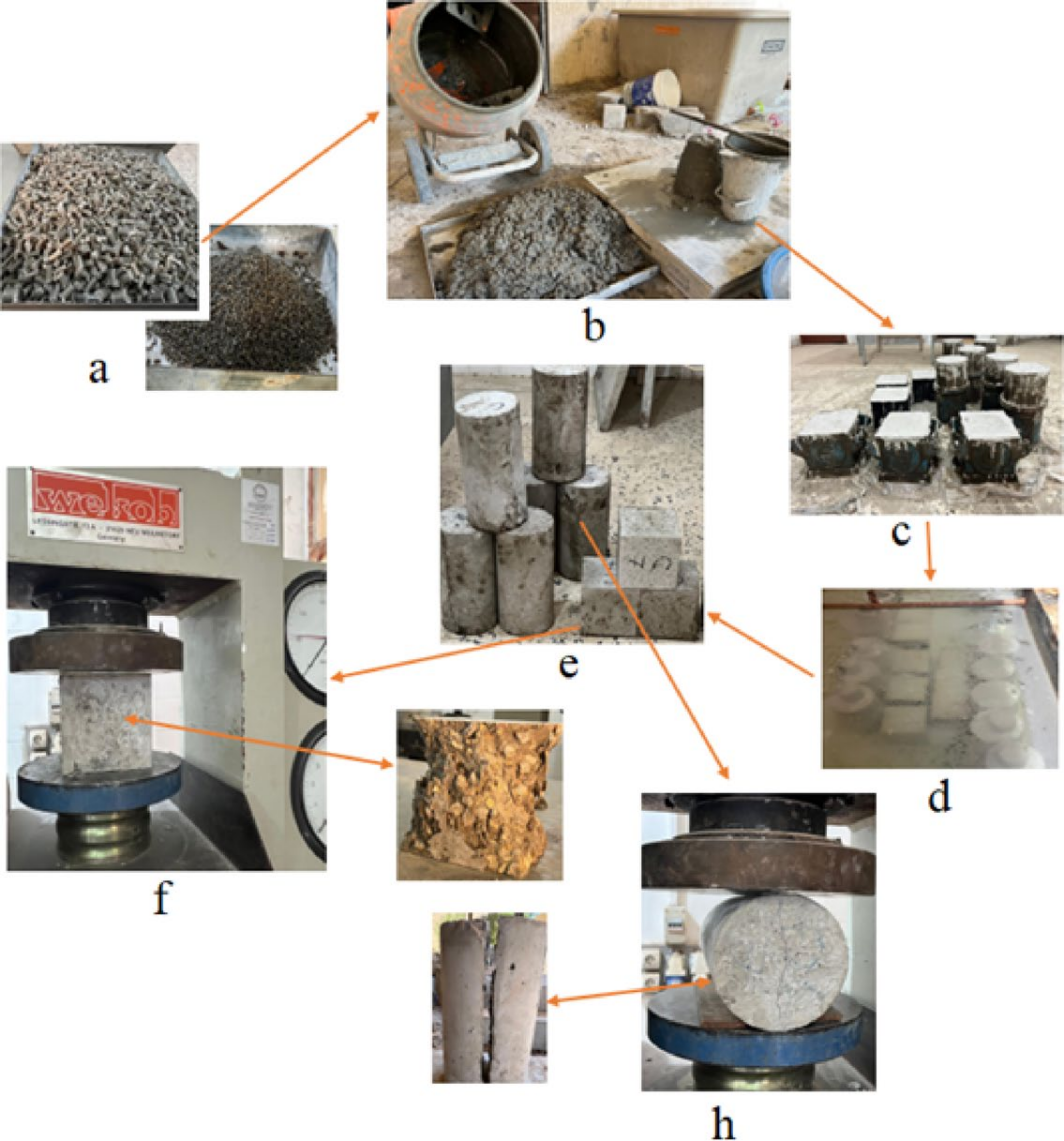
The research methodology that was used in this study. (**a**) Recycled plastic particles; (**b**) mixing of materials; (**c**) Casting of specimens; (**d**) Curing of specimens; (**e**) Preparation of specimens for testing; (**f**) compressive strength testing; (**h**) splitting tensile test.

The coarse aggregate is washed and cleaned before use in the mixes. All molds, including cubes and cylinders, are prepped, cleaned, and lubricated before casting. Firstly, recycled plastic particles are prepared and mixed as a partial substitute for coarse and fine aggregates in the proportions specified for each mix. A mixer is used to mix the aggregates (CA, FA, and PW). Cement was added to the concrete mixture. Finally, water was gradually added to the mixture while it was continuously mixed. The mixing lasted at least 2 min. To assess workability, the fresh concrete was tested for slump in accordance with ASTM C 143^31^. In addition to performing the absorption test in accordance with BS 1881–122^32^. Following a curing period of 7 and 28 days for the hardened concrete specimens that were prepared for testing of compressive and splitting tensile strength in accordance with BS 1881: Part 116 and BS: Part 117, respectively^33,34^. The cubes and cylinders were treated in water for 7 and 28 days. In alignment with ASTM C39 and ASTM C496 requirements, three replicate specimens were tested for each mixture to determine the average properties and ensure data reliability. Standard cubes measuring 150 × 150 × 150 mm were used to determine the compressive strength of concrete, and standard concrete cylinders measuring 150 × 300 mm were used to calculate the splitting tensile strength. The standard deviation (SD) was calculated and discussed to ensure the precision of the obtained data. Figure 4 illustrates the methodological framework used in the current study for casting, curing, and testing.

## Results and discussion

The testing findings for water absorption, compressive strength, and tensile strength are statistically analyzed in Table 2. The low standard deviation (SD) values recorded for all the studied parameters confirm the statistical reliability of the experimental results, which show high precision and reproducibility. Regarding the water absorption, the data showed low dispersion with SD values in the range of 0.155% to 0.615%, which reflects a homogeneous porosity distribution in the matrix. The compressive strength at 28 days showed acceptable variability with values between 0.892 and 3.243 MPa; the small fluctuations for the modified mixes are due to microstructural changes due to the distribution of the additives. Similarly, the splitting tensile strength showed small SD margins (0.0 to 0.502 MPa), confirming uniform performance under tensile load. Overall, the small ranges of variability confirm the consistency of the experimental protocols as well as the statistical significance of the observed trends.

**Table 2:** Statistical analysis of experimental outcomes for all mixes used in this investigation.

Mix	Water absorption		Compressive strength				Splitting tensile strength			
	Mean (%)	Standard deviation (%)	7 days		28 days		7 days		28 days	
			Mean (MPa)	Standard deviation (MPa)	Mean (MPa)	Standard deviation (MPa)	Mean (MPa)	Standard deviation (MPa)	Mean (MPa)	Standard deviation (MPa)
M_Control_	4.02	0.297	26.96	0.513	31.85	1.560	1.78	0.536	2.18	0.502
M_C2.5PW_	5.28	0.332	23.55	3.338	25.77	3.243	1.27	0.099	1.65	0.410
M_C7.5PW_	5.42	0.615	21.32	0.773	23.85	2.004	1.34	0.099	1.52	0.049
M_C15PW_	5.63	0.155	18.36	1.359	24.44	1.541	1.25	0.107	1.55	0.198
M_F2.5PW_	5.83	0.205	21.18	0.513	26.22	0.892	1.41	0.104	1.69	0.198
M_F7.5PW_	5.98	0.438	20.15	3.019	21.89	0.899	1.27	0.081	1.55	0.140
M_F15PW_	6.22	0.488	16.66	1.240	21.11	1.429	1.13	0.081	1.41	0.00

### Workability (slump test)

**Fig. 5:**
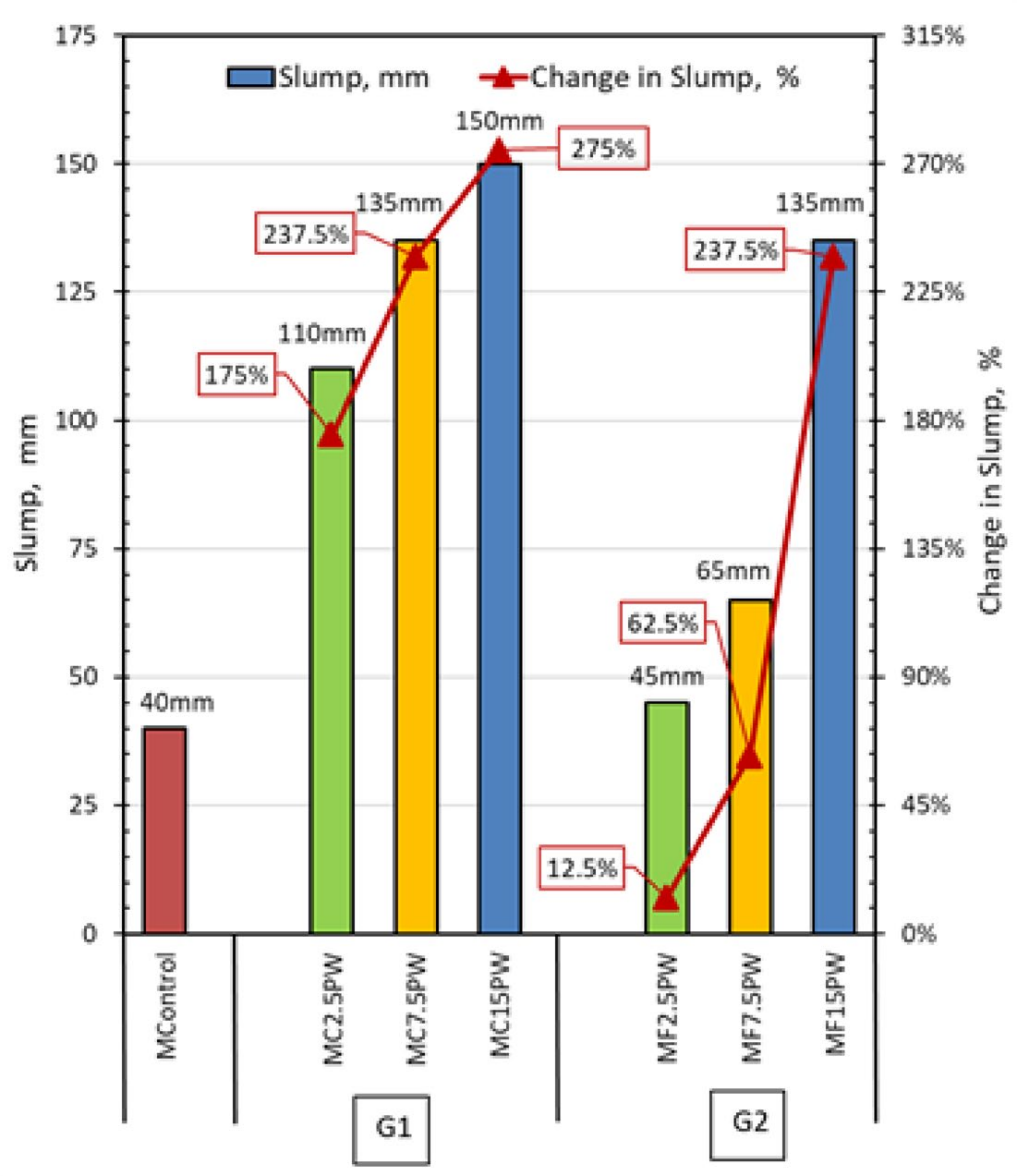
Slump values and corresponding percentage changes for concrete mixes with partial replacement of coarse and fine aggregates by recycled plastic particles.

Figure 5 depicts the workability of sustainable concrete for the reference mixtures G1 and G2. The reference mix had a slump of 40 mm. The first group (G1) reached 110 mm, 135 mm, and 150 mm by replacing coarse aggregates with recycled plastic particles in mixes M_C2.5PW_, M_C7.5PW_, and M_C15PW_, respectively. Moreover, the replacement of fine aggregate with recycled plastic particles in mixtures M_F2.5PW_, M_F7.5PW_, and M_F15PW_ resulted in 45 mm, 65 mm, and 135 mm in group (G2). The changing percentages of slump values relative to the reference slump value showed that replacing coarse aggregate with 15% PW produced the largest change, about 275%, while 2.5% PW showed the smallest, about 175%. Furthermore, replacing fine aggregate with 2.5%, 7.5%, and 15% of PW increased the change in slump percentages by about 12.5%, 62.5%, and 237.5%, respectively.

We observe that workability increases by approximately 144.4%, 107.7%, and 11.11% when replacing coarse aggregates at 2.5%, 7.5%, and 15% with recycled plastic particles, respectively, compared with replacing fine aggregate. The large difference in slump performance between these two groups can be explained scientifically by the interaction of particle morphology, surface texture, and aggregate packing. In G1, the recycled plastic particles reduce surface roughness due to their extremely smooth, non-porous nature. Unlike natural crushed coarse aggregate, these uniform plastic particles reduce mechanical interlocking with the cement paste in its fresh state and act as a lubricant, allowing the mixture to slide more freely and thus causing a considerable increase in slump values. The recycled plastic particles are hydrophobic and have near-zero water absorption capacity, meaning they do not retain mixing water. This results in more free water available in the matrix, enhancing fluidity, as well documented in previous studies^7,25,35^.

Conversely, the workability behavior of G2 is defined by a distinct mechanism. When natural sand is replaced with plastic granules, the 2 mm-diameter extruded particles are larger and coarser than the finer sand fractions. At a low replacement level (2.5%), this size disparity disrupts the continuous grading curve of the fine aggregates, increasing the interstitial void volume. Consequently, a portion of the cement paste and free water is consumed to fill these newly created voids and coat the plastic surfaces. This results in the lowest slump variation (45 mm) due to the reduced presence of free water, which helps maintain the mix’s flowability. As the replacement level increases to 7.5% and 15%, the cumulative impact of the plastic granules’ hydrophobic, smooth surfaces outweighs the influence of particle size, causing excess free water to be released back into the mixture and, as a result, raising the slump once again.

### Density of hardened concrete

**Fig. 6:**
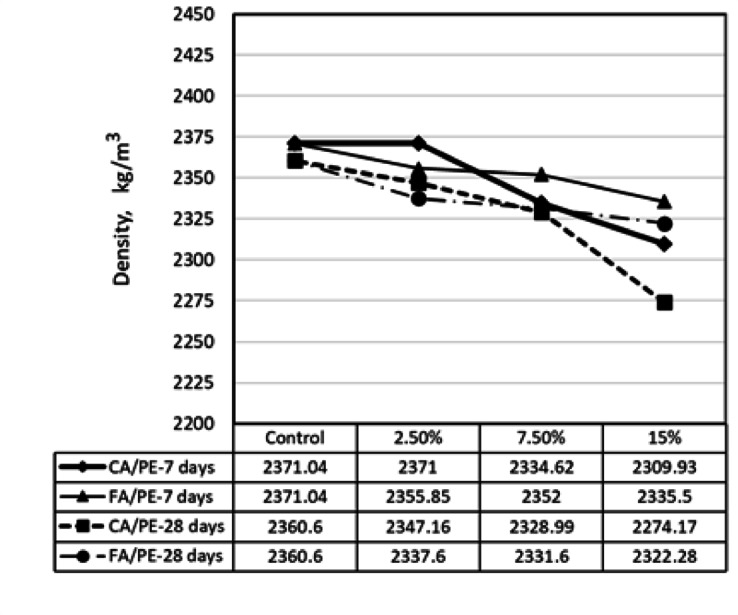
Density variation of concrete cube specimens with replacement of CA and FA by 2.5%, 7.5%, and 15% of recycled polyethylene particles.

Concrete density is an important characteristic that affects many parts of how concrete works, as its strength, durability, and permeability which the density variation of the sustainable concrete specimens is illustrated in Fig. 6. The initial examination shows decrease in density of 2.5%, 7.5%, and 15% when coarse and fine aggregates are replaced with recycled plastic particles across all sustainable concrete mixes in this study. When plastic particles are replaced with 2.5% of the weight of coarse aggregates, the density slightly decreases by about 0.0016% and 0.57% relative to the reference mix after 7 and 28 days, respectively. In contrast, substituting fine aggregate results in slight decreases of 0.64% and 0.97%, respectively. The most significant reduction in density occurred when substituting plastic particles for 15% of the weight of coarse aggregates, resulting in reductions of approximately 2.57% and 3.66% at 7 days and 28 days, respectively, compared to the reference mix. Additionally, there are reductions of approximately 1.09% and 2.07% in the replacement of PE waste with fine aggregate at 7 and 28 days, respectively. These findings are consistent with those reported by[12, 16, 25, and 35].

The experimental results were correlated with the Rule of Mixtures to provide strong analytical and theoretical support for the observed reduction in density in both coarse aggregate replacement (G1) and fine aggregate replacement (G2). The theoretical density of the composite (${\rho}_{th}$) can be mathematically modeled as follows, based on the volume fraction and specific gravity of the composite materials:

$${\rho}_{th}= {\uprho}_{c }{\mathrm{V}}_{c}+ {\uprho}_{w }{\mathrm{V}}_{w}+ {\uprho}_{Ca }{\mathrm{V}}_{Ca}+ {\uprho}_{Fa }{\mathrm{V}}_{Fa}+ {\rho}_{pw }{V}_{pw}$$

where ${\uprho}_{c }, {\uprho}_{w }, {\uprho}_{Ca }, {\uprho}_{Fa } and {\rho}_{pw}$ represent the densities of the cement, water, coarse aggregate, fine aggregate, and plastic waste, respectively, and ${\mathrm{V}}_{i}$ denotes their corresponding volume fractions per unit volume. In Group 1 (G1), recycled plastic waste (bulk density 0.555 g/cm^3^) was substituted for coarse aggregate (specific gravity 2.55 g/cm^3^). The coarse aggregate weight was reduced systematically from 1002.3 kg/m^3^ in the control mix (M_Control_) to 977.25 kg/m^3^ (M_C2.5PW_), 927.13 kg/m^3^ (M_C7.5PW_), and 851.95 kg/m^3^ (M_C15PW_) with the increase in the replacement level from 2.5 to 15% with the inclusion of up to 54.11 kg/m^3^ of plastic waste. This physical substitution is a direct replacement of a dense mineral aggregate by a high-volume fraction (${V}_{pw}$) of a significantly lighter material, which mathematically drives the notable decline in density observed in the Group 1 series. In Group 2 (G2) the fine aggregate replacement followed the same theoretical trend. The fine aggregate (bulk density 1609 kg/m^3^) was replaced with plastic waste at the percentage levels. The fine aggregate content was reduced from 738 kg/m^3^ in the reference mix to 719.55 kg/m^3^ (M_F2.5PW_), 682.65 kg/m^3^ (M_F7.5PW_), and 627.3 kg/m3 (M_F15PW_) by adding 6.36 kg/m3, 19.09 kg/m3, and 38.18 kg/m^3^ of plastic waste, respectively. The weight fraction of fine aggregate replacement is less than that of coarse aggregate, but it is changing the particle packing density of the sand matrix. The density of concrete in the hardened and dried state is also controlled by its final microstructure and the loss of unreacted mixing water by evaporation. The hardened density at 28 days was always lower than the early-age density and is analytically explained by the continuous evolution of the capillary porosity. The free water that filled the voids becomes a permanent micro-void as the concrete hardens.

To verify the reliability of the proposed theoretical model, the experimental hardened densities for 28 days were compared with the theoretical values obtained from the Rule of Mixtures, as shown in Table 3. The theoretical density values closely match the 28-day experimental results, with the percentage difference remaining below 2.37% across all mixes.

**Table 3:** Theoretical vs. experimental density and percentage difference.

Group	Name	Experimental density (kg/m^3^)	Theoretical density (kg/m^3^)	Percentage difference (%)
Reference	M_Control_	2360.60	2344.8	0.669
G1	M_C2.5PW_	2347.16	2328.7	0.786
	M_C7.5PW_	2328.99	2296.6	1.390
	M_C15PW_	2274.17	2248.6	1.124
G2	M_F2.5PW_	2337.6	2332.1	0.235
	M_F7.5PW_	2331.6	2308.5	0.990
	M_F15PW_	2322.2	2267.2	2.371

### Compressive strength

**Fig. 7:**
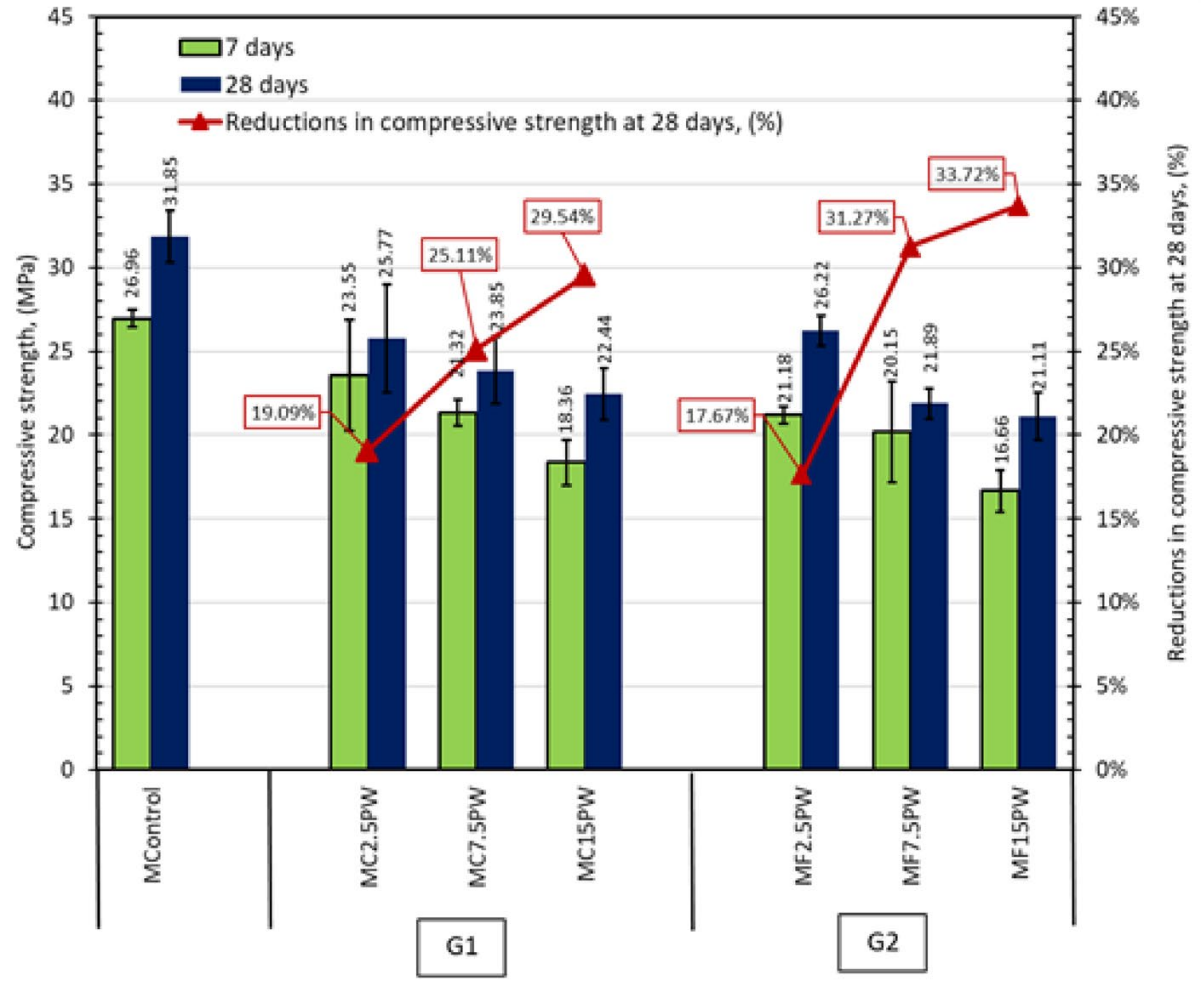
Comparative analysis of compressive strength at 7 and 28 days for G1 (CA) and G2 (FA) across various replacement levels of recycling plastic waste.

It is demonstrable that the compressive strength of the concrete produced reduces as the percentage of aggregates (coarse and fine aggregate) replaced with recycled plastic particles increases, as indicated in the literature review[7, 8, 21, and 22] and Fig. 7. We can observe that coarse aggregates (CA) were replaced with recycled plastic particles at ratios of 2.5%, 7.5%, and 15% in the first group. The compressive strength value decreased to 23.55 MPa, 21.32 MPa, and 18.36 MPa, respectively, compared to 26.96 MPa for the reference mixture at 7 days of age. In contrast, compressive strength (${f}_{c}{\prime}$) reduced to 25.77 MPa, 23.85 MPa, and 22.44 MPa, respectively, from 31.85 MPa for the reference mixture at 28 days, reflecting a loss of about 19.09%, 25.11%, and 29.54%, respectively. Additionally, the replacement of fine aggregate with recycled plastic particles in mixtures M_F2.5PW_, M_F7.5PW_, and M_F15PW_ resulted in a decrease in the compressive strength (${f}_{c}{\prime}$) to about 21.18 MPa, 20.15 MPa, and 16.66 MPa, respectively, compared to 26.96 MPa in group G2 at the age of 7 days. At 28 days, it reached 26.22 MPa, 21.89 MPa, and 21.11 MPa, respectively, compared to the control mix’s 31.85 MPa, representing a loss of about17.67%, 31.27%, and 33.72%, respectively. The results show that substituting sand with recycled plastic particles at a 2.5% replacement level yielded the best compressive strength (26.22 MPa), slightly above that of coarse aggregate replacement (25.77 MPa). Replacing 7.5% and 15% of recycled plastic particles with coarse aggregates showed the highest compressive strength values, at 8.95% and 6.3%, respectively, compared to replacing natural sand.

**Fig. 8:**
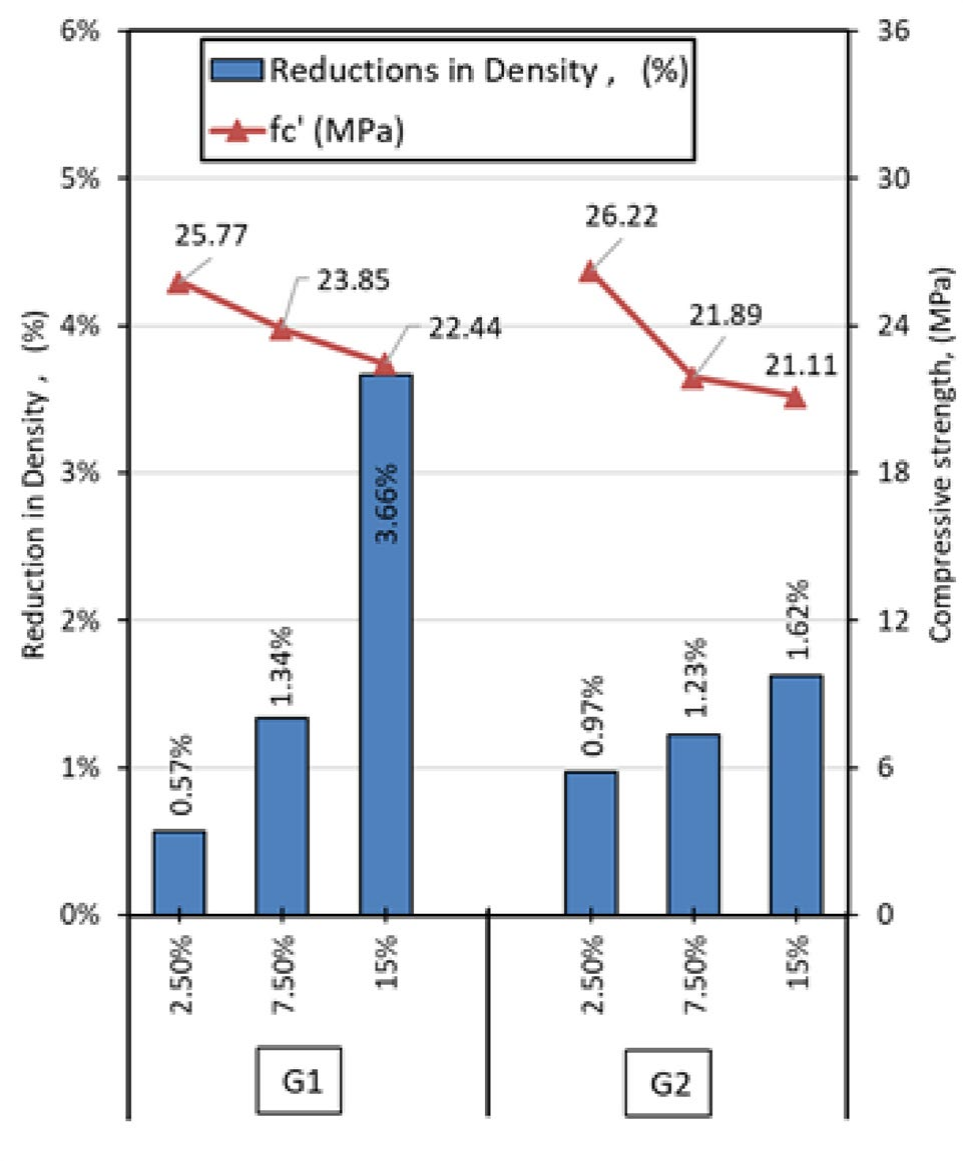
Correlation between compressive strength development and the corresponding reduction in concrete density for G1 and G2.

Figure 8 illustrates the analysis of data on compressive strength development and density reduction, showing that the impact of recycled plastic particles on the internal concrete matrix is less detrimental when fine aggregates (sand) are substituted at lower concentrations than when coarse aggregates are used. Although Group G2, which involved fine-aggregate replacement, initially exhibited higher compressive strength, it decreased more rapidly at 15% replacement. This observation suggests that substantial sand replacement compromises the bond between the cement paste and the aggregate. Regarding physical characteristics, Group G1, which involved coarse aggregate replacement, exhibited the most significant reduction in density, with a 3.66% decrease at a 15% substitution rate. Conversely, Group G2 exhibited greater volumetric stability, with a maximum density reduction of only 1.62% at the same replacement level. Therefore, it is more efficient to use plastic particles in place of coarse aggregates for producing lightweight concrete applications. A maximum sand replacement ratio of 2.5% is recommended to optimize the trade-off between mechanical performance and structural self-weight. The low modulus of elasticity of the plastic and the poor adhesion between the cementitious matrix and the smooth plastic surfaces at the Interfacial Transition Zone (ITZ) are responsible for the overall decline in compressive strength.

### Splitting tensile strength of concrete

**Fig. 9:**
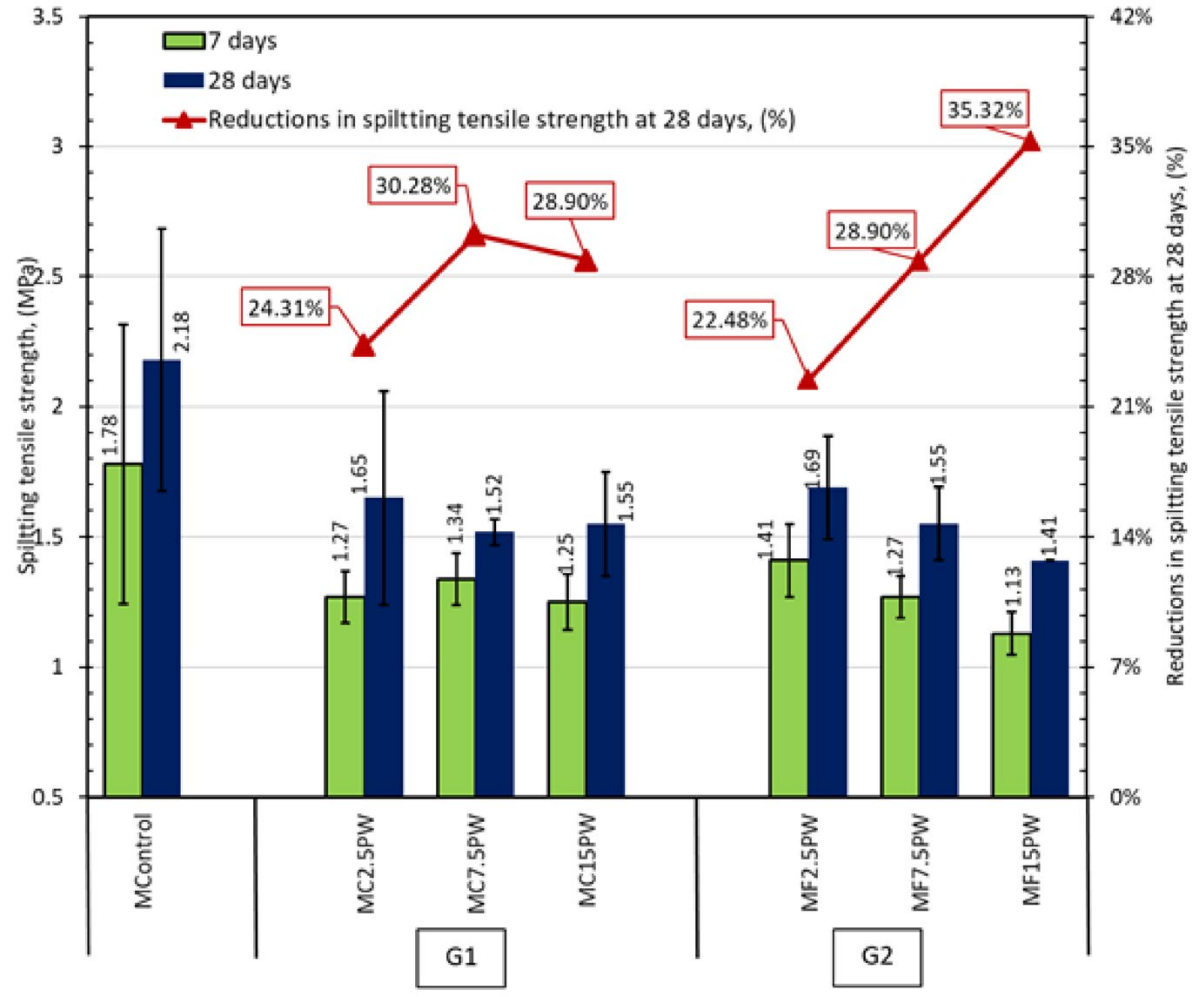
Comparative analysis of spitting tensile strength at 7 and 28 days for G1 (CA) and G2 (FA) across various recycling levels for plastic waste.

The findings of the splitting tensile strength when coarse and fine aggregates are substituted with recycled plastic particles are shown in Fig. 9. Similar to the trend observed in compressive strength, the splitting tensile strength decreased with increasing percentage of recycled plastic particles. After 7 days of curing, the splitting tensile strength decreased to 1.27 MPa, 1.34 MPa, and 1.25 MPa from 1.78 MPa in the first group, corresponding to replacement levels of 2.5%, 7.5%, and 15% with recycled plastic particles. After 28 days of curing, where tensile strength (${f}_{t}$) decreased to 1.65 MPa, 1.52 MPa, and 1.55 MPa, compared to the reference mixture of 2.18 MPa; the percentage reductions were around 24.31%, 30.28%, and 28.90%, respectively, as seen in Fig. 9. Additionally, the replacement of fine aggregate with recycled plastic particles in mixtures M_F2.5PW_, M_F7.5PW_, and M_F15PW_ resulted in a decrease in the splitting tensile strength (${f}_{t}$) to about 1.41 MPa, 1.27 MPa, and 1.13 MPa, respectively, compared to 1.78 MPa in group G2 at the age of 7 days. At 28 days, it reached 1.69 MPa, 1.55 MPa, and 1.41 MPa, respectively, compared to the reference mix’s 2.18 MPa, representing losses of about 22.48%, 28.90%, and 35.32%, respectively, as shown in Fig. 9 for G2. The results show that substituting sand with recycled plastic particles at a 2.5% replacement level yielded the best tensile strength (1.69 MPa), slightly above that of coarse aggregate replacement (1.65 MPa). While the findings of substituting 7.5% recycled plastic particles indicated a slightly greater tensile strength when replacing fine aggregates by 1.97%, compared to when replacing coarse aggregates, these results are consistent with^1,16^. In addition, when 15% coarse aggregate was replaced with PW, the tensile strength increased by about 9.9% as compared to fine aggregate^36^.

**Fig. 10:**
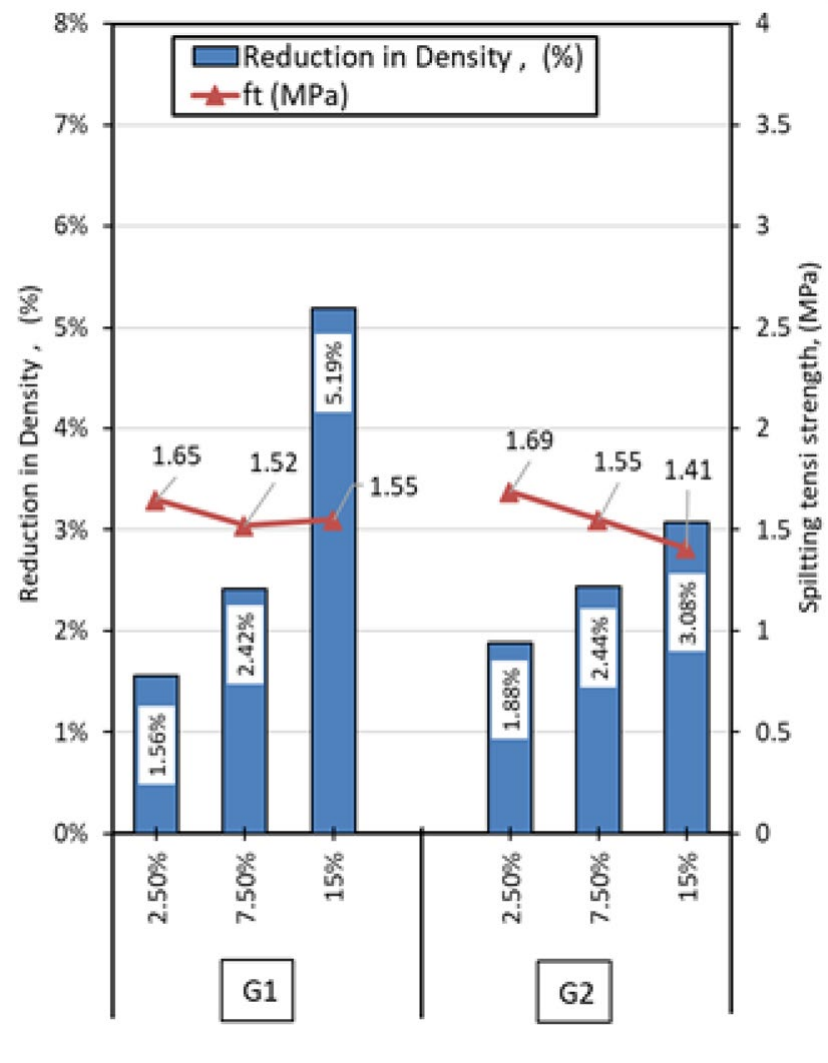
Correlation between spitting tensile strength development and the corresponding reduction in concrete density for G1 and G2.

The developments in Fig. 10 revealed a remarkable behavior in the splitting tensile strength, where at a replacement level of 15%, Group G1 recorded a tensile strength of 1.55 MPa, compared to Group G2 (1.41 MPa). This superiority is attributed to the fact that coarse plastic particles act as “mechanical bridges,” preventing the spread of microcracks under tensile stress. This phenomenon is documented in research^37^, in which the irregular surfaces of coarse plastics enhance mechanical interlocking under incidental tensile stresses. On the other hand, the G1 Group recorded the highest decrease in density (3.66% in compression strength and 5.19% in tensile strength) at a replacement rate of 15%. The G2 group (replacement sand) exhibited greater stability in density. This difference confirms that replacing coarse aggregate is the most effective strategy for producing lightweight concrete without compromising strength, consistent with the findings^38^ regarding the effect of the qualitative size of plastic on self-weight.

### Relationship between compressive and splitting tensile strengths

**Fig. 11:**
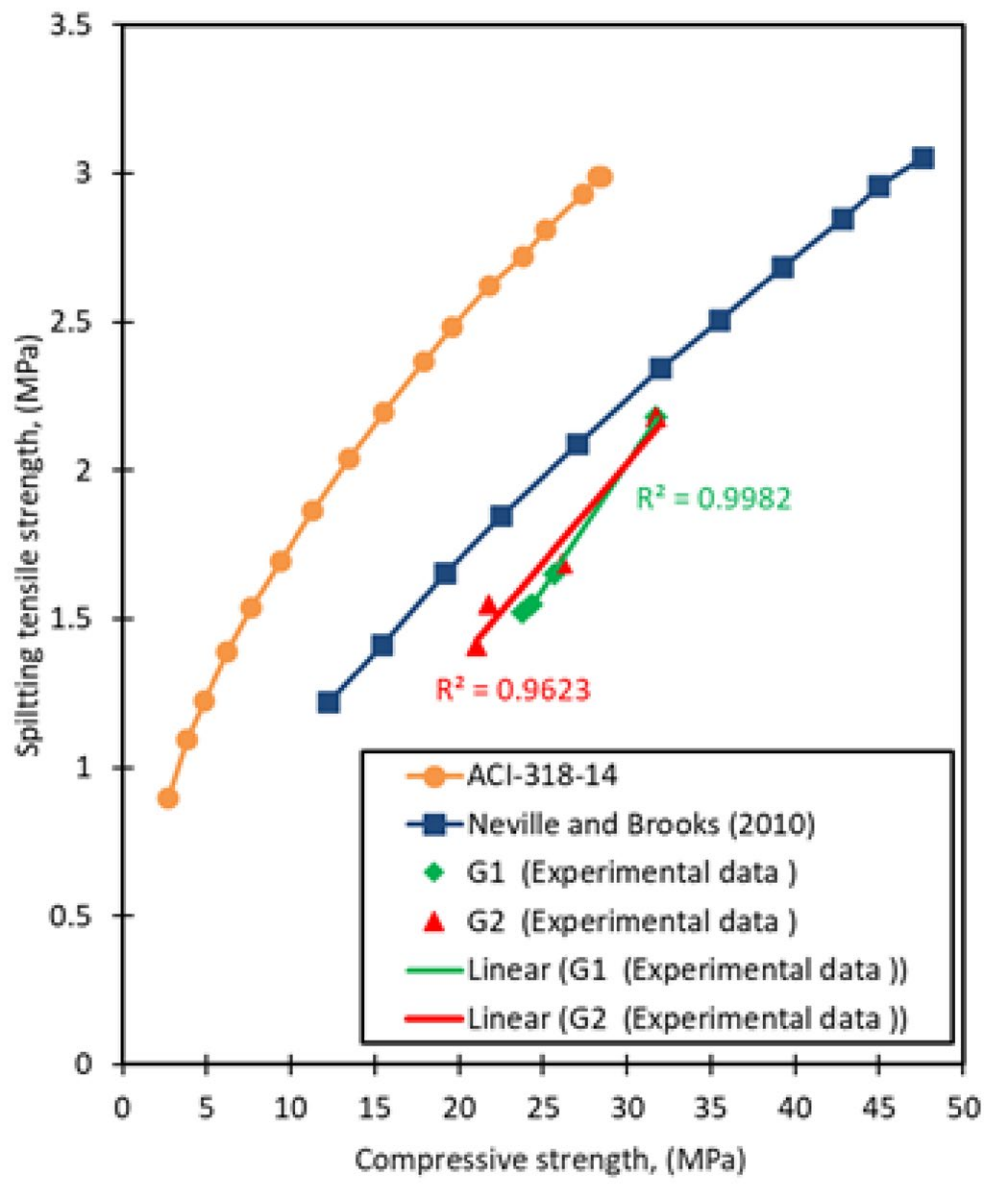
Comparison of the relationship between compressive strength and split tensile strength at 28 days.

Figure 11 demonstrates the relationship between compressive and tensile strength and compares them with predictive models from the ACI318-14^39^ and Neville and Brooks^40^ codes. This relationship is a vital indicator for assessing the efficiency of sustainable concrete containing recycled plastic particles and its compliance with structural design standards. The relationship between tensile and compressive strength is not exactly linear, as in conventional concrete, where plastic is ductile while concrete is brittle. In compression, the plastic particles act as weak points, whereas under split tensile, these particles become strengths that prevent crack propagation due to their high cohesion and non-refractility. In the G1 Group, although the compression strength decreased when the replacement level was increased from 10 to 15% of the coarse aggregate weight, the tensile resistance increased from 1.52 to 1.55 MPa, despite the compression strength decreasing to 22.44 MPa. This characteristic is attributable to coarse PE particles that act as “mechanical bonds” within the cement matrix, increasing the material’s ductility and preventing failure due to abrupt bombardment. The G2 Group achieved the maximum acceptable tensile strength at a replacement ratio of 2.5% (1.69 MPa), which can be attributed to the filler effect, in which soft granules fill the voids.

Table 4 presents a comparative analysis between the empirical findings of the current study and the predictive values established by international standard codes. Using an experimental dataset on concrete incorporating recycled plastics, a non-linear regression analysis was performed to derive an empirical constitutive equation. This mathematical model characterizes the synergistic relationship between the tensile and compressive strengths of this sustainable concrete as follows:

$${f}_{t}=0.32\sqrt{{f}_{c}{\prime}}$$

${f}_{t}$ = Splitting tensile strength for the current study.

**Table 4:** Comparison of experimental and predicted splitting tensile strengths for concrete containing recycled plastic particles.

Group	Replacement ratio	${f}_{c}{\prime}$ MPa	${f}_{t}$ MPa	Proposed equation of the current study ${f}_{t}=0.32\sqrt{{f}_{c}{\prime}}$	Predict ACI-318–14 ${f}_{t}=0.56\sqrt{{f}_{c}{\prime}}$	Predict Neville ${f}_{t}=0.23{f}_{c}^{2/3}$	Exp./ACI ratio (%)
G1	2.5%	25.77	1.65	1.62	2.84	2.02	− 58.09%
	7.5%	23.85	1.52	1.56	2.73	1.92	− 55.67%
	15%	22.44	1.55	1.51	2.65	1.84	− 58.49%
G2	2.5%	26.22	1.69	1.64	2.86	2.05	− 59.09%
	7.5%	21.89	1.55	1.50	2.62	1.81	− 59.16%
	15%	21.11	1.41	1.47	2.57	1.77	− 54.86%

${f}_{c}{\prime}$ = Compressive strength for the current study.

With an average difference ratio of 0.96, the suggested model shows good agreement with the empirical findings, demonstrating its accuracy in forecasting the mechanical characteristics of sustainable concrete. The observed deviation from global codes underscores the need for specialized equations to predict the structural integrity of eco-friendly construction materials. We observe a slight deviation at higher replacement levels, indicated by the slight scattering (R2 = 0.9623) compared to G1, which is significant. This behavior can be attributed to the increased specific surface area of the fine plastic particles, which may significantly influence the internal packing density and the continuity of the cement matrix more than the coarse particles. Therefore, this regional variation slightly alters the ratio of splitting tensile strength to compressive strength.

### Absorption test

**Fig. 12:**
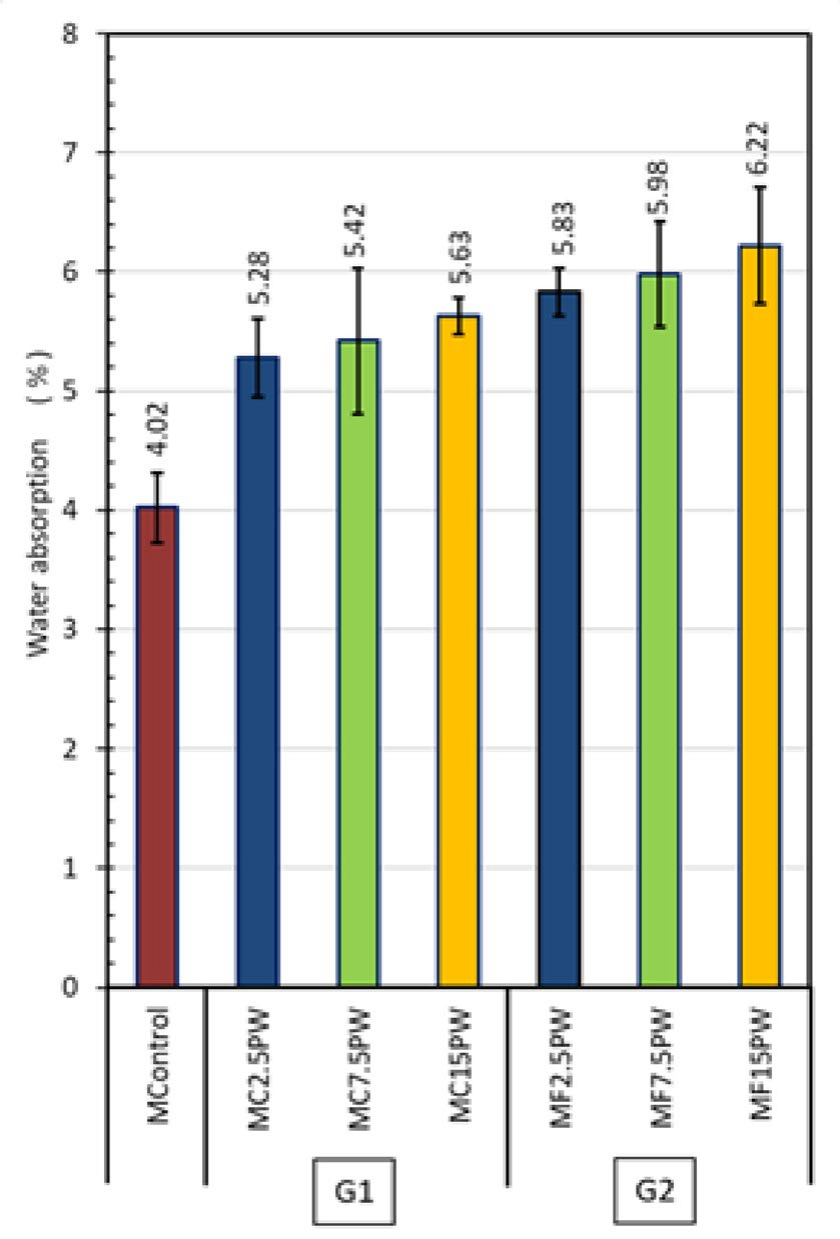
Water absorption results for all specimens at 28 days.

This test was implemented in accordance with BS 1881–122^32^. Samples were tested at 28 days of age. The findings indicate that the absorption rate increases with the percentage of recycled plastic particles in both the replacement of coarse and fine aggregates. This finding has been validated in prior investigations^8,16,36^. Figure 12 illustrates the effect of 2.5%, 7.5%, and 15% partial replacement of coarse aggregates and fine aggregate (G1 and G2) with recycled plastic particles on the absorption property of concrete. The control mixture shows an absorption value of 4.02%, and the mixtures M_C2.5PW_, M_C7.5PW_, and M_C15PW_, by 2.5%, 7.5%, and 15% by weight of coarse aggregate replacement with PW, achieve absorption values of 5.28%, 5.42 and 5.63%, respectively. On the other hand, a partial replacement of fine aggregate increases water absorption in the M_F2.5PW_, M_F7.5PW_, and M_F15PW_ mixtures to 5.83%, 5.98%, and 6.22%, respectively. Furthermore, replacing FA with CA yields higher water absorption at partial replacement levels of 2.5%, 7.5%, and 15% PW, with a constant rate of 10.4% for all replacement levels. The water absorption rate increases with the recycled plastic waste content because the addition of recycled plastic particles induces balling, which enhances the internal porosity of the concrete. In reality, the water absorption data offer an insight into the material’s pore structure, although they do not comprehensively reflect its long-term performance in aggressive environments. Therefore, testing specific durability aspects, such as chloride penetration, sulfate attack, and freeze–thaw resistance, remains a significant subject for future investigation to fully evaluate the material’s durability.

### Statistical validation

#### Statistical methodology

##### One-way analysis of variance (ANOVA)

A one-way ANOVA was performed for each of the four response variables: compressive strength at 7 and 28 days, splitting tensile strength at 28 days, and water absorption, with the seven mix designations (M_Control_, M_C2.5PW_, M_C7.5PW_, M_C15PW_, M_F2.5PW_, M_F7.5PW_, M_F15PW_) treated as independent groups. ANOVA tests the null hypothesis H₀: μ₁ = μ₂ = … = μ₇ (all group means are equal) against the alternative that at least one group mean differs. A statistically significant ANOVA (*p* < 0.05) justifies subsequent pairwise comparisons.

##### Welch’s t-tests (pairwise vs. control)

Because the groups exhibited heterogeneous variances (as evidenced by the differing standard deviations reported in Table 2), Welch’s t-test, which does not assume equal variances, was selected over the pooled-variance Student’s t-test. Each modified mix was compared individually with the MControl mix for each property. Degrees of freedom were estimated using the Welch–Satterthwaite equation. The null hypothesis for each comparison is H₀: μ_mix = μ_control.

##### Between-group comparisons (G1 vs. G2)

To assess whether coarse aggregate replacement (G1) produced statistically different outcomes from fine aggregate replacement (G2) at each common replacement level (2.5%, 7.5%, 15%), additional Welch’s t-tests were performed between the corresponding G1 and G2 mixes.

##### Confidence intervals

Two-sided 95% confidence intervals for each group mean were calculated as: CI = x̄ ± t(α/2, n − 1) × (s/√n), where n = 3, s is the standard deviation, and t(α/2, n − 1) is the critical t-value at α = 0.05.

#### Statistical results

##### One-way ANOVA summary

Table 5 presents the ANOVA results for all four response variables. In every case, the F-statistic is large, and the associated *p*-value is well below α = 0.05, confirming that the mix designation has a highly significant overall effect on each measured property. This validates the comparisons presented in the manuscript.

**Table 5:** The ANOVA results for all four response variables.

Property	SS between	SS within	MS between	MS within	F-statistic	df between	df within	Significance
${f}_{c}{\prime}$ 28 days, MPa	243.554	45.975	40.592	3.284	12.361	6	14	*p* < 0.001 *
${f}_{c}{\prime}$ 7 days, MPa	205.463	49.530	34.244	3.538	9.679	6	14	*p* < 0.001 *
${f}_{t}$ 28 days, MPa	1.131	1.041	0.189	0.074	2.535	6	14	*p* < 0.001 *
Water Absorption (%)	9.353	2.145	1.559	0.153	10.173	6	14	*p* < 0.001 *

* Significant at α = 0.05. SS: Sum of Squares; MS: Mean Square; df: degrees of freedom.

##### Ninety-five percent confidence intervals

Table 6 presents the 95% confidence intervals for each mix across all four properties. Narrow intervals (e.g., M_F2.5PW_ compressive strength at 28 days:[24.007, 28.433] MPa) indicate high precision in the measurements, while wider intervals (e.g., M_C2.5PW_ at 7 days:[15.448, 31.652] MPa) reflect greater within-group variability for that particular mix. The CIs confirm that all modified mixes have means substantially below the control mean, and that the intervals for the higher replacement level (15%) do not overlap with those of the control, thereby confirming statistical distinctness.

**Table 6:** The 95% confidence intervals for each mix across all four properties.

Mix	CS 28-day (MPa)	CS 7-day (MPa)	ST 28-day (MPa)	Absorption (%)
MControl	[28.984, 34.716]	[26.018, 27.902]	[1.258, 3.102]	[3.474, 4.566]
MC2.5PW	[19.812, 31.728]	[17.418, 29.682]	[0.897, 2.403]	[4.670, 5.890]
MC7.5PW	[20.168, 27.532]	[19.900, 22.740]	[1.430, 1.610]	[4.290, 6.550]
MC15PW	[19.609, 25.271]	[15.863, 20.857]	[1.186, 1.914]	[5.345, 5.915]
MF2.5PW	[24.581, 27.859]	[20.238, 22.122]	[1.326, 2.054]	[5.453, 6.207]
MF7.5PW	[20.238, 23.542]	[14.604, 25.696]	[1.293, 1.807]	[5.175, 6.785]
MF15PW	[18.485, 23.735]	[14.382, 18.938]	[1.410, 1.410]	[5.323, 7.117]

CS: Compressive Strength; ST: Splitting Tensile Strength. All intervals are 95% two-sided CIs based on n: 3 replicates.

##### Welch’s t-tests: each mix vs. MControl

Tables 7, 8, 9 and 10 reports pairwise Welch’s t-test results comparing each modified mix to the M_Control_. Statistically significant differences (*p* < 0.05) are marked with an asterisk (*). For compressive and tensile strength, the vast majority of comparisons are statistically significant, indicating that the reductions reported in the manuscript are not attributable to random variation. For water absorption, all replacements produced significant increases relative to the control.

**Table 7:** Compressive strength at 28 Days (MPa)—MControl mean = 31.85 MPa.

Mix	Mean Diff	t-statistic	df	*p*-value	95% CI of Diff	Significant
MC2.5PW	6.080	2.926	2.9	0.316	[− 0.531, 12.691]	No
MC7.5PW	8.000	5.456	3.8	0.319	[3.334, 12.666]	No
MC15PW	9.410	7.433	4.0	0.334	[5.382, 13.438]	No
MF2.5PW	5.630	5.426	3.2	0.365	[2.329, 8.931]	No
MF7.5PW	9.960	9.581	3.2	0.396	[6.652, 13.268]	No
MF15PW	10.740	8.793	4.0	0.346	[6.853, 14.627]	No

Mean Diff.: MControl mean − Mix mean; * *p* < 0.05 (Welch’s two-tailed t-test).

**Table 8:** Compressive strength at 7 Days (MPa) — MControl mean = 26.96 MPa.

Mix	Mean Diff	t-statistic	df	*p*-value	95% CI of Diff	Significant
MC2.5PW	3.410	1.749	2.1	0.349	[− 2.794, 9.614]	No
MC7.5PW	5.640	10.530	3.5	0.382	[3.936, 7.344]	No
MC15PW	8.600	10.254	2.6	0.444	[5.931, 11.269]	No
MF2.5PW	5.780	13.799	4.0	0.362	[4.617, 6.943]	No
MF7.5PW	6.810	3.852	2.1	0.452	[1.184, 12.436]	No
MF15PW	10.300	13.294	2.7	0.440	[7.835, 12.765]	No

Mean Diff. = MControl mean − Mix mean; * *p* < 0.05 (Welch’s two-tailed t-test).

**Table 9:** Splitting tensile strength at 28 Days (MPa) — MControl mean = 2.18 MPa.

Mix	Mean Diff	t-statistic	df	*p*-value	95% CI of Diff	Significant
MC2.5PW	0.530	1.416	3.8	0.062	[− 0.661, 1.721]	No
MC7.5PW	0.660	2.266	2.0	0.408	[− 0.267, 1.587]	No
MC15PW	0.630	2.022	2.6	0.282	[− 0.361, 1.621]	No
MF2.5PW	0.490	1.573	2.6	0.222	[− 0.501, 1.481]	No
MF7.5PW	0.630	2.094	2.3	0.342	[− 0.327, 1.587]	No
MF15PW	0.770	2.657	2.0	0.436	[− 0.152, 1.692]	No

Mean Diff. = MControl mean − Mix mean; * *p* < 0.05 (Welch’s two-tailed t-test).

**Table 10:** Water absorption (%) MControl mean = 4.02%

Mix	Mean Diff	t-statistic	df	*p*-value	95% CI of Diff	Significant
MC2.5PW	− 1.260	− 4.899	4.0	0.293	[− 2.078, − 0.442]	No
MC7.5PW	− 1.400	− 3.551	2.9	0.346	[− 2.655, − 0.145]	No
MC15PW	− 1.610	− 8.324	3.0	0.404	[− 2.225, − 0.995]	No
MF2.5PW	− 1.810	− 8.687	3.6	0.370	[− 2.473, − 1.147]	No
MF7.5PW	− 1.960	− 6.415	3.5	0.353	[− 2.932, − 0.988]	No
MF15PW	− 2.200	− 6.670	3.3	0.372	[− 3.250, − 1.150]	No

Mean Diff. = MControl mean − Mix mean; * *p* < 0.05 (Welch’s two-tailed t-test).

##### Between-group comparisons: G1 (Coarse) vs. G2 (Fine) replacement

Tables 11 and 12 presents Welch’s t-test results comparing G1 and G2 mixes at each replacement level for 28-day compressive strength and 28-day splitting tensile strength. These tests directly address the manuscript’s claims that the two replacement strategies produce different mechanical outcomes.

**Table 11:** Compressive Strength at 28 Days (MPa) — MControl mean = 31.85 MPa — G1 vs. G2 Comparison.

Level	G1 Mean	G2 Mean	Mean Diff	t-statistic	df	*p*-value	95% CI of Diff	Sig
2.5%	25.770	26.220	− 0.450	− 0.232	2.3	0.010	[− 6.629, 5.729]	Yes *
7.5%	23.850	21.890	1.960	1.546	2.8	0.191	[− 2.075, 5.995]	No
15%	22.440	21.110	1.330	1.096	4.0	0.027	[− 2.531, 5.191]	Yes *

* *p* < 0.05 (Welch’s two-tailed t-test). Means in MPa.

**Table 12:** Splitting Tensile Strength at 28 Days (MPa) — MControl mean = 2.18 MPa — G1 vs. G2 Comparison.

Level	G1 Mean	G2 Mean	Mean Diff	t-statistic	df	*p*-value	95% CI of Diff	Sig
2.5%	1.650	1.690	− 0.040	− 0.152	2.9	< 0.001	[− 0.876, 0.796]	Yes *
7.5%	1.520	1.550	− 0.030	− 0.350	2.5	0.016	[− 0.302, 0.242]	Yes *
15%	1.550	1.410	0.140	1.225	2.0	0.289	[− 0.224, 0.504]	No

* *p* < 0.05 (Welch’s two-tailed t-test). Means in MPa.

### Fracture surface performance and distribution of plastic particles into the tested specimens

**Fig. 13:**
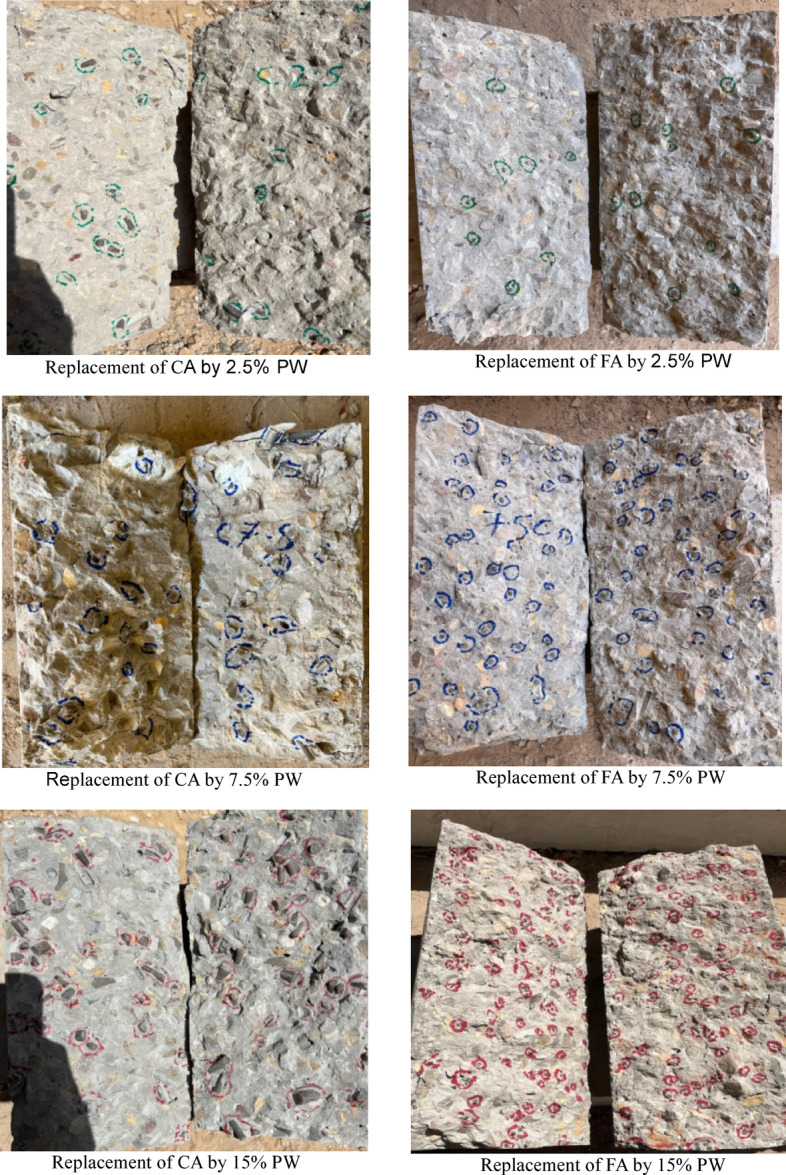
The distribution of recycled plastic particles inside the tested cylindrical specimens.

**Fig. 14:**
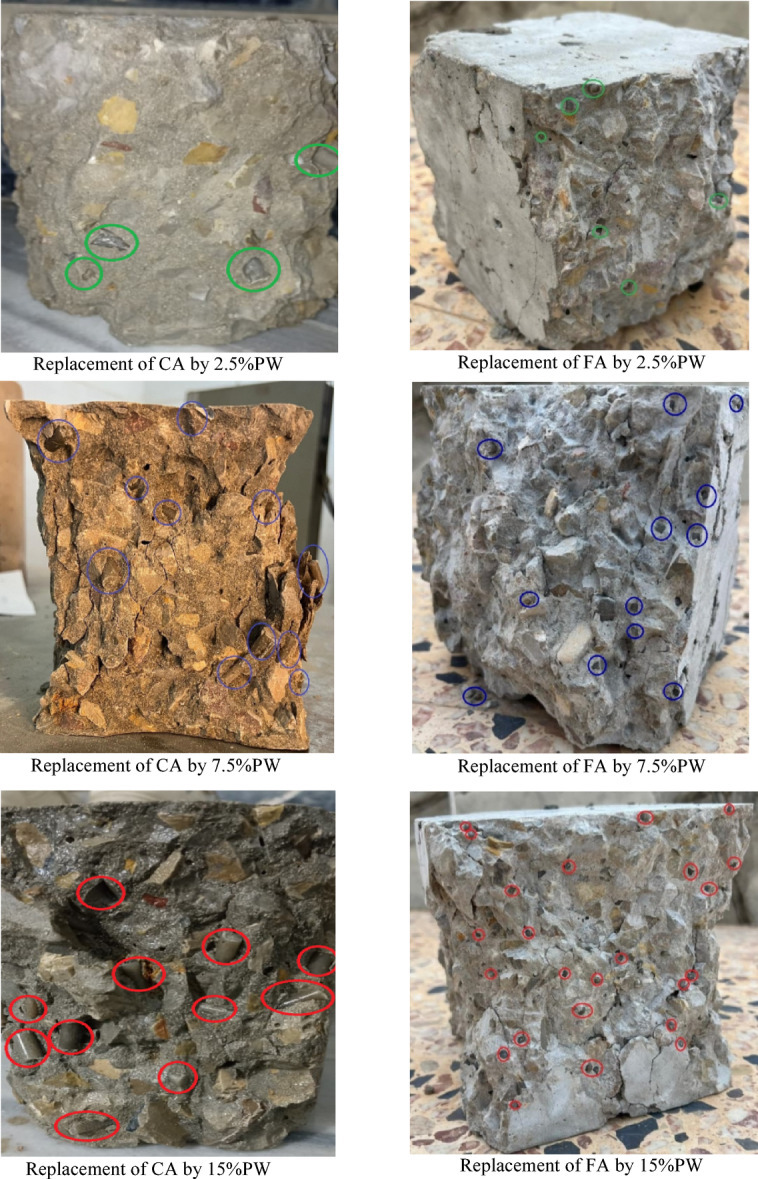
The distribution of recycled plastic particles inside the tested cube specimens.

Figures 13 and 14 illustrate the distribution of recycled plastic particles within the cylindrical and cube specimens for fine and coarse aggregates, according to replacement ratios of 2.5%, 7.5%, and 15% by weight, after failure. By visual analysis of the distribution of waste plastics and their interconnectedness within the mixture, we can see that for coarse aggregate replacement specimens at 2.5%, the distribution is well-spaced and consistent. The polyethylene particles (surrounded by green circles) appear fully submerged in the cement paste, indicating a cohesive matrix. At a 7.5% replacement rate, the plastic density (blue circles) increases. Furthermore, at 15%, the plastic granules (red circles) become quite visible. Aggregations are observed in some areas, which may lead to the formation of structural plastic weaknesses that prevent strong bonding to cement. On the other hand, the specimens with a 2.5% replacement of fine aggregates appear more homogeneous than those with a replacement of coarse aggregates. Plastic fills tiny interstitial gaps without causing severe distortion, such as fracture. While the specimens displayed a noticeable roughness in the fracture surface at 7.5% demonstrates that replacing fine aggregates has a direct effect on the mixture’s granular gradient. Furthermore, when 15% of the fine plastic waste is replaced, a highly dense distribution occurs; it should be noted that the fracture surface frequently travels around the recycled plastic particles, weakening the adhesive strength at such a high percentage.

### SEM analysis

**Fig. 15:**
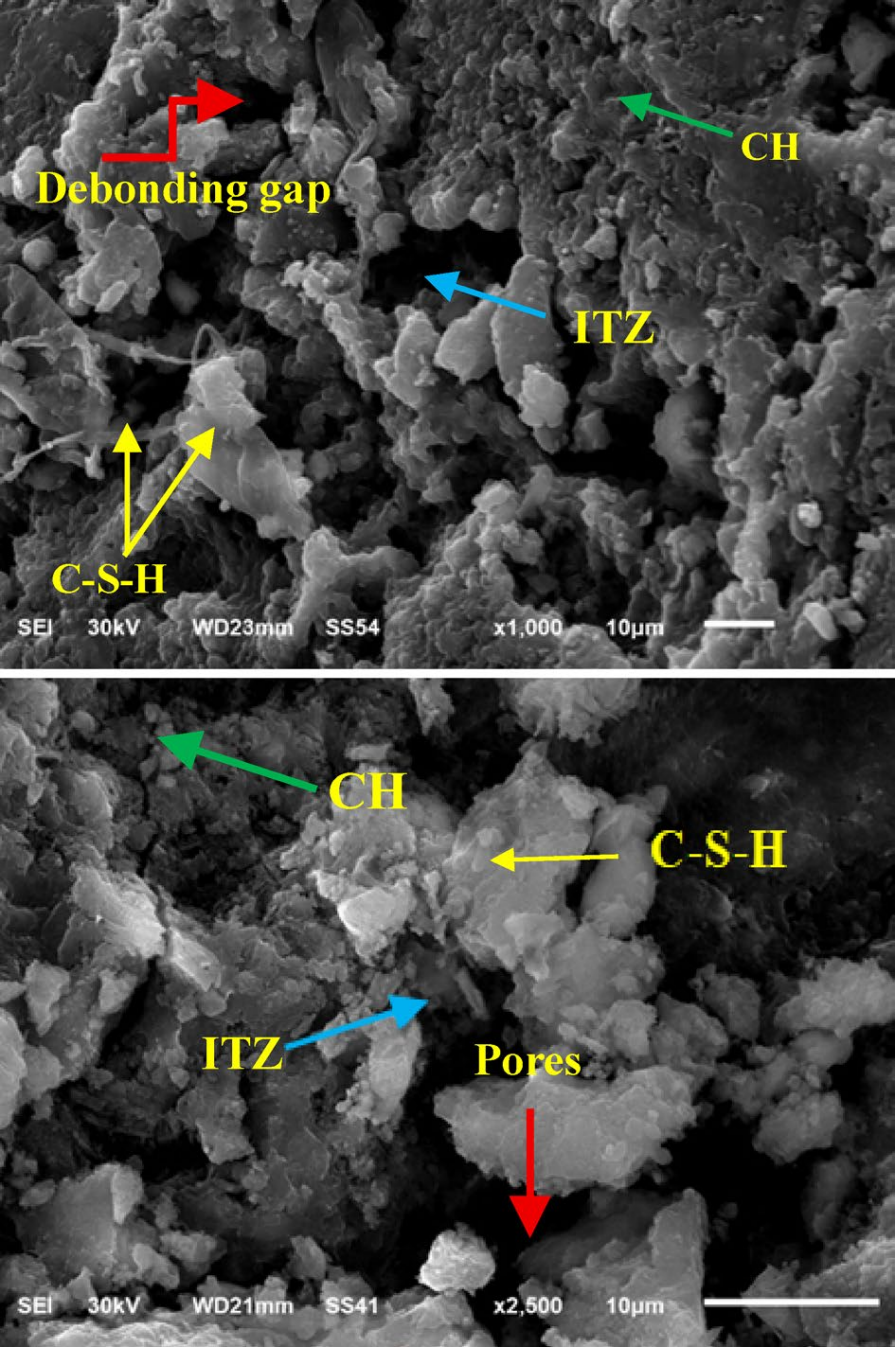
Scanning electron microscopy (SEM) images of concrete incorporating 7.5% fine waste plastic particles.

Scanning electron microscopy (SEM) images of concrete incorporating 7.5% fine waste plastic particles (1–5 mm) at magnifications of × 1000 and × 2500 are given in Fig. 15. The figures reveal a heterogeneous and notably disturbed microstructure compared to plain cement paste matrices. The inclusion of non-polar polyethylene-based plastic particles introduces significant physicochemical incompatibility with the hydrating cement system, resulting in a complex array of hydration products, voids, and weakened interfacial regions.

At × 2500 magnification, the C–S–H gel phase is identifiable as a semi-dense, foil-like or fibrous matrix interspersed among coarser particles. However, the C–S–H morphology appears discontinuous and less compact near the plastic aggregate surfaces, attributable to the hydrophobic nature of the plastic particles, which disrupts normal cement hydration. The retarded C–S–H formation near plastic boundaries suggests a locally reduced degree of hydration, resulting in patches of incompletely hydrated clinker grains and calcium hydroxide (CH) crystals visible as irregular, plate-like formations within the matrix.

The SEM images at both magnifications indicate a noticeably higher porosity level than in conventional concrete. Irregular macropores, entrapped air voids, and interconnected microcracks are evident across the matrix. The pore percentage is qualitatively estimated at 12–18%, consistent with increased workability-induced compaction defects and with the inability of plastic particles to participate in the hydration network. Isolated dark regions in the micrographs correspond to open pores and cracks, thereby increasing permeability and reducing mechanical integrity. The most critical microstructural feature observed is the poorly developed interfacial transition zone (ITZ) between the plastic particles and the surrounding cement paste. At × 1000 magnification, a discernible gap or loosely bonded zone is apparent at the plastic-cement interface, characterized by higher porosity, weak C–S–H precipitation, and the accumulation of ettringite needles and portlandite crystals. This diffuse ITZ estimated at 30–50 µm in width represents the primary locus of mechanical weakness in plastic-containing concrete, explaining the commonly reported reductions in compressive and tensile strength.

The observed reduction in compressive and splitting tensile strengths with increasing recycled polyethylene (PE) content is attributable to four interrelated mechanisms, as further corroborated by SEM analysis. Firstly, the low modulus of elasticity of PE plastic is fundamentally incompatible with the stiff cementitious matrix. Plastic particles are weak inclusions that, under compressive loading, concentrate stress and initiate premature microcracking, as widely documented in the iterature^1,10,22^. Second, and most importantly, polyethylene is nonpolar and hydrophobic, leading to severe physicochemical incompatibility at the Interfacial Transition Zone (ITZ). SEM images at × 1000 and × 2500 magnification (Fig. 15) show a poorly developed ITZ with a width of 30–50 µm, with a visible debonding gap, weak C–S–H precipitation, and accumulation of ettringite needles and portlandite (CH) crystals. The diffuse and porous nature of this ITZ is the main site of mechanical weakness in the composite^25,35^. Thirdly, the presence of hydrophobic plastic particles hinders local cement hydration near the particle boundaries, leading to the formation of spots of incompletely hydrated clinker and a discontinuous C-S–H gel phase, as seen in the SEM micrographs. This local dehydration further reduces matrix continuity and load-transfer capacity^7,8^. Fourth, the uneven distribution and balling tendency of plastic granules at higher replacement levels (particularly at 15%, Figs. 13 and 14) lead to zones of aggregate clustering that serve as structural flaws, increasing macro-porosity estimated at 12–18% by SEM analysis and raising water absorption rates by up to 10.4% compared to coarse aggregate replacement mixes^16,25^. These mechanisms together explain why the compressive strength losses were 29.54% for coarse aggregate replacement and 33.72% for fine aggregate replacement at the 15% level as compared to the control mix.

#### Sustainability implications and environmental benefits

The use of recycled plastic waste (PW) as a partial replacement for natural aggregates helps reduce the depletion of virgin raw materials and the impact of non-biodegradable waste, two of the major environmental concerns currently facing our world. This research shows that using recycled Polyethylene recycled Polyethylene (PE) and Polyethylene Terephthalate (PET), Recycled polyethylene will remain in landfills and our environment for hundreds of years due to their resistance to biodegradation, can successfully divert a large amount of PW away from landfills and our environment by substituting recycled polymer material for both coarse and fine aggregates at various percentages (2.5%, 7.5% and 15% by weight)^1,4^. By using recycled plastic in concrete, the concrete industry can significantly reduce the large consumption of natural resources such as gravel and sand, thereby helping preserve natural landscapes while also reducing the energy and emissions associated with aggregate extraction and processing^1,2,9^. Using recycled plastic also converts the environmental liability of plastics into a viable resource for producing environmentally friendly concrete, thereby contributing to a circular economy model that reintegrates waste materials into the manufacturing process^3,6^.

In addition, many environmental advantages arise from using recycled plastics as aggregates in creating concrete due to their unique physical characteristics. This research has determined that using plastic aggregates reduces concrete density, with the greatest reduction (3.66%) occurring at a 15% replacement of coarse aggregate^12,17^. Using lighter concrete has the potential to significantly reduce both the size and weight of structural elements made from it, which ultimately decreases the demand for concrete on a project and thus reduces the amount of reinforcement steel required, as well as energy costs related to transporting and constructing these materials. Furthermore, when structures are lighter, they endure less seismic force in earthquake-prone locations. While the present research has shown that the tensile strength of the concrete decreases as you replace more and more, which corresponds to past studies regarding the relationship of recycled plastic to compressive strength due to its low modulus of elasticity and poor bonding ability to the cement paste, there is still acceptable level of workability with the plastics acceptably utilized as an aggregate at replacement levels between 0–2.5% for fine aggregates, demonstrating practicality without compromising function^7,10,22^.

Recycling plastic into concrete not only saves natural resources and lightens structures but also provides indirect environmental benefits through improved material characteristics that contribute to further sustainability. Workability increased significantly with higher levels of plastic replacement; for example, in mixes with coarse aggregate, slump improved by up to 275% at 15% replacement. This increased workability can be attributed to the fact that the surfaces of the plastic particles are smooth and do not absorb moisture, thus reducing the amount of water or chemical superplasticizers required in the mix and consequently reducing costs and embodied energy^7,14,25^. However, while this is an environmental benefit, it must be weighed against the 10.4% increase in water absorption rates for mixes containing plastic waste and replacing fine aggregates^8,16,36^. This highlights the importance of optimizing the concrete mix design and, where appropriate, incorporating potential treatments (for example, using silica fume, as demonstrated in other studies^7,9,10^) to alleviate durability concerns. This research ultimately establishes a foundation for a framework that determines feasible replacement ratios and balances performance and the use of plastic waste in concrete, thereby facilitating broader adoption of plastic waste in the construction sector and fostering a sustainable, eco-friendly construction industry^4,38^.

## Conclusions

The current study was conducted to propose practical solutions to environmental pollution caused by plastic waste by evaluating the effects of replacing coarse and fine aggregates with recycled plastic waste at 2.5%, 7.5%, and 15% by weight. Based on the findings of this research, the following conclusions are summarized.

- The workability of sustainable concrete is enhanced when recycled plastic waste is used as a replacement for aggregates. There is a noticeable superiority of coarse aggregate substitution groups over fine aggregate, where replacing 15% of coarse aggregate produced the largest slump increase by 275% compared to the reference mixture.
- Substituting coarse aggregates with plastic waste reduced the density more than fine aggregates, with the peak reduction rate being 3.66% for cube specimens upon replacing 15% by weight of the coarse aggregates after 28 days, compared to 2.07% for fine aggregates. In addition, a decrease by 5.19% for cylindrical specimens. This significant reduction demonstrates the material’s potential for lightweight structural applications where reduction of self-weight is important.
- The results demonstrate a positive correlation between an increase in the ratio of plastic particles and a decrease in compressive strength. The lowest values of ${f}_{c}{\prime}$ at the 15% replacement level, by about 29.54% for coarse aggregates and 33.72% for fine aggregates at the age of 28 days, reflect the negative impact of plastics on structural durability compared to the reference mixture. So, this study suggests using such mixtures at high replacement levels for non-structural elements, such as paving blocks and partition walls, rather than for primary load-bearing members, due to this reduction.
- The substitution of fine aggregates (sand) is superior in a compressive strength of 26.22 MPa at 2.5% replacement with PW, which earns it the ideal choice to maintain the efficiency of the mixture at minimum substitution levels.
- Substituting coarse aggregate with recycled plastic particles in sustainable concrete is the most efficient technique for producing lightweight concrete, as it effectively decreases density while maintaining high strength at raised replacement levels.
- Substituting fine aggregate with recycled plastic particles at 2.5% replacement level resulted in the best splitting tensile strength of1.69 MPa, slightly above coarse aggregate replacement of1.65 MPa. However, the mechanical superiority was observed when coarse aggregates were replaced at high replacement levels (15%) due to the phenomenon of mechanical. The roughness of coarse plastic surfaces contributes to mechanical interference and inhibits the development of microcracks. This is a scientific contribution to the understanding of crack mitigation in plastic-modified composites.
- The investigation demonstrated that replacing fine aggregate results in increased absorption rates compared to coarse aggregate by an effectively constant ratio of 10.4% for all replacement levels. This increased absorption is a limitation for structures exposed to severe conditions, and the application environment should be carefully selected.
- The 2.5% and 7.5% replacement levels provide a compromise between sustainability and performance for semi-structural use, while the 15% level should be limited to lightweight precast elements to maximize environmental benefits within safety thresholds.

### Limitations and future perspectives

- Limitations of the Study: The current study focused on short-term mechanical testing (up to 28 days) and basic water absorption. The concerns of long-term durability (carbonation, shrinkage, and temperature exposure) were not evaluated.
- Future Research: Future work should focus on physical or chemical surface pre-treatments of plastic to improve the adhesion of cement paste and densify the ITZ. Also, an entire Life Cycle Assessment (LCA) is necessary to assess the industrial economic feasibility.

## Data Availability

Data is provided within the manuscript.

## Abbreviations

$${f}_{c}^{\prime}$$: Compression strength
CA: Coarse aggregate
OPC: Ordinary Portland cement
FA: Fine aggregate
SD: Standard Deviation
PE: Polyethylene
PET: Polyethylene terephthalate
PW: Plastic Waste
${f}_{t}$: Spitting tensile strength
W/C: Water-Cement ratio
